# Analysis of codon usage patterns in *Ginkgo biloba* reveals codon usage tendency from A/U-ending to G/C-ending

**DOI:** 10.1038/srep35927

**Published:** 2016-11-03

**Authors:** Bing He, Hui Dong, Cong Jiang, Fuliang Cao, Shentong Tao, Li-an Xu

**Affiliations:** 1 Co-Innovation Center for Sustainable Forestry in Southern China, Nanjing Forestry University, Nanjing 210037, China; 2 Department of Botany and Plant Pathology, Purdue University, West Lafayette, IN 47907, USA; 3 State Key Laboratory of Crop Stress Biology for Arid Areas, Northwest A&F University, Yangling, Shanxi 712100, China

## Abstract

As one of the most ancient tree species, the codon usage pattern analysis of *Ginkgo biloba* is a useful way to understand its evolutionary and genetic mechanisms. Several studies have been conducted on angiosperms, but seldom on gymnosperms. Based on RNA-Seq data of the *G. biloba* transcriptome, amount to 17,579 unigenes longer than 300 bp were selected and analyzed from 68,547 candidates. The codon usage pattern tended towards more frequently use of A/U-ending codons, which showed an obvious gradient progressing from gymnosperms to dicots to monocots. Meanwhile, analysis of high/low-expression unigenes revealed that high-expression unigenes tended to use G/C-ending codons together with more codon usage bias. Variation of unigenes with different functions suggested that unigenes involving in environment adaptation use G/C-ending codons more frequently with more usage bias, and these results were consistent with the conclusion that the formation of *G. biloba* codon usage bias was dominated by natural selection.

Triplet codons play vital roles in all biological organisms as the basic units for the encoding of mRNA. Since the genetic code is degenerate, most amino acids except methionine and tryptophan, are encoded by 2–6 different codons^1^. Genes have tendency in the choice (i.e. non-random use) of synonymous codons to encode amino acids, which is known as codon usage bias. Analysis of codon usage bias provides a clue to reveal laws of genetics and evolution^2^, design degenerate primers and study appropriate external expression systems^3^. A recent study reported that codon usage bias was able to affect the expression, structure and function of protein^4^ and the local rate of translation elongation as well^5^. Furthermore, codon usage bias was shown to be closely associated with the molecular mechanism of translation^6^, new genes discovery^7^, sex determination^8^ and other biological functions^9^.

One of the most representative theories to explain codon usage bias is selection-mutation-drift, proposing that codon usage bias is mainly affected by mutation pressure, genetic drift and natural selection^10^,^11^. Additionally, gene expression level^12^ and gene length^13^,^14^ were found to be correlated with the formation of codon usage bias. Nevertheless the main forces of codon usage bias vary greatly in different species. The genic GC content is a very important feature in the analysis of codon usage bias, and the GC contents at the third base of one codon (GC3) are considered to most likely directly reflect codon usage pattern^15^. Previous studies indicated that dicots and monocots tended to use A/U and C/G as ending codons, respectively^16^.

*Ginkgo biloba* L. is endemic to China and is the only known member of the Ginkgopsida^17^. As one of the most ancient seed plants, the earliest *Ginkgo*-like trees can be traced to approximately 280 million years ago^18^. There are still many debates on the detailed classification of gymnosperms, and *G. biloba* plays a very important role in this classification system due to its unique characteristics, such as the rare broad leaves and diclinism, which are very different to other gymnosperms^19^. Although studies on codon usage bias have been widely studied in *Caenorhabditis*^20^, *Arabidopsis*^21^, *Populus*^22^, *Myrica rubra*^23^ and *Bombyx mori*^24^—no systematic research on codon usage patterns and the related base composition (GC3 contents) together with functional classification in gymnosperms has been reported. In our previous study, we performed RNA-Seq sequencing of *G. biloba* and the data (accession number: SRP062414) was made publicly available^25^. Analysis of the codon usage patterns in *G. biloba* will show its codon usage bias and enable determining which factor was the dominant force in its formation. In addition, whether *G. biloba* shows some differentiation in codon usage pattern involved in different metabolic pathways need to be further investigated. Here, we systematically studied the codon usage pattern in *G. biloba* and evaluated the effects of various factors. The results might provide an explanation or a reference for studies on the genetic evolution mechanism of *G. biloba.*

## Results

### Base composition of *G. biloba* genes indicates A/U bias

**Figure 1:**
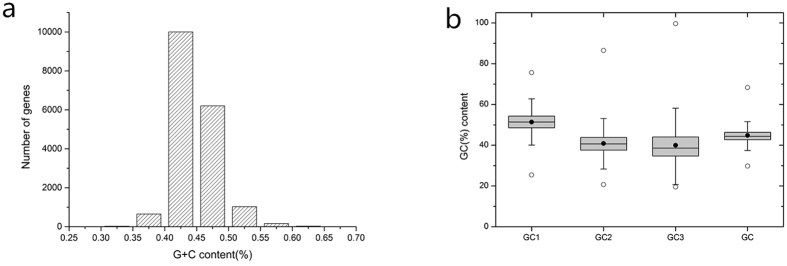
(**a**) Distribution of G + C contents in *G. biloba* unigenes. (**b**)Box plot of *G. biloba* GC content variation in different coding positions. The dark dots represent the mean, the bottom and top of the box were the lower and upper quartiles, respectively, and the ends of the whiskers were the lowest and highest data still within 1.5 times the interquartile range of the lower and higher quartiles, respectively.

The transcriptome of *G. biloba* was sequenced based on the Illumina Hiseq2000 high-throughput platform, and Trinity was used for assembly. There was 25.08G of data obtained, including more than 185 million raw reads, 112,946 transcripts and 68,547 unigenes. The average length of transcripts was 1,262 bp. A total of 17,579 unigenes were selected after the screening procedure for further analysis of codon usage pattern. Their GC contents were in the range of 29.3–68.3%, and the average value was 44.81% (Fig. 1a). Most unigenes had GC contents <50%, and the number of unigenes with GC contents of 40.0–45.0% was the largest group (n = 10,004). GC1 had the highest mean value of 51.39% (SD = 4.43), followed by GC2 (mean = 40.88%, SD = 5.17) and GC3 (mean = 39.02%, SD = 7.45). Although the mean value of GC3 was the lowest, it had the widest distribution range of 0.195–0.996, suggesting that codon usage bias in *G. biloba* may vary widely according to different unigenes and the overall pattern may not be highly conserved^26^ (Fig. 1b). The mean GC content of *G. biloba* untranslated region (UTR) was 37.89% which was slightly lower than mean GC3 content. For a better understanding of *G. biloba* base composition, we estimated the overall GC content of the *G. biloba* genomic data that was sequenced previously in our laboratory and derived a mean value was 35.68% (unpublished data). This indicated that there might be GC enrichment in *G. biloba* coding sequences relative to its UTR and noncoding regions. To further investigate this assumption, 100 full-length gene sequences longer than 500 bp were randomly selected for *Pinus taeda* and *Picea abies* from the NCBI database and similar rules were found (Supplementary File 1).

### Synonymous codon usage pattern in *G. biloba*

The relative synonymous codon usage (RSCU) values of *G. biloba* unigenes are shown in Table 1. Among all 59 codons, 28 were defined as preferred codons. The RSCU value of AGA encoding for arginine (Arg) was the highest (2.04) and this was the only RSCU >2.0. There were 19 codons with RSCU >1.2 among the other 27 preferred codons. There were also 23 codons ending with A/U among all 28 preferred codons, suggesting that generally *G. biloba* tend to have A/U-ending codons, and that base composition differences arising from natural selection or mutation pressure in *G. biloba* might play a decisive role in the codon usage bias^10^.

The average effective number of codons (ENC) value of *G. biloba* was 52.66 and the maximum was 61 (SD = 8.21), suggesting that the overall codon usage bias was not very extreme. The three minimum ENC values were 24.91, 25.68 and 25.86; according to the NCBI non-redundant protein sequence (Nr) database annotation, they encoded ribosomal proteins (24.91 and 25.68) and glutathione transferase (25.86) respectively. In general, a gene is thought to possess strong codon bias if its ENC <36^24^,^27^. It is noteworthy that 47 unigenes encoding ribosomal proteins were identified in addition to 17,579 nuclear unigenes. Those nuclear unigenes encoding for ribosomal proteins showed higher mean GC contents (50.61%) and lower mean ENC values (48.12), and these patterns differed from *G. biloba* nuclear unigenes.

### ENC–GC3 analysis suggests many factors play important roles

**Figure 2:**
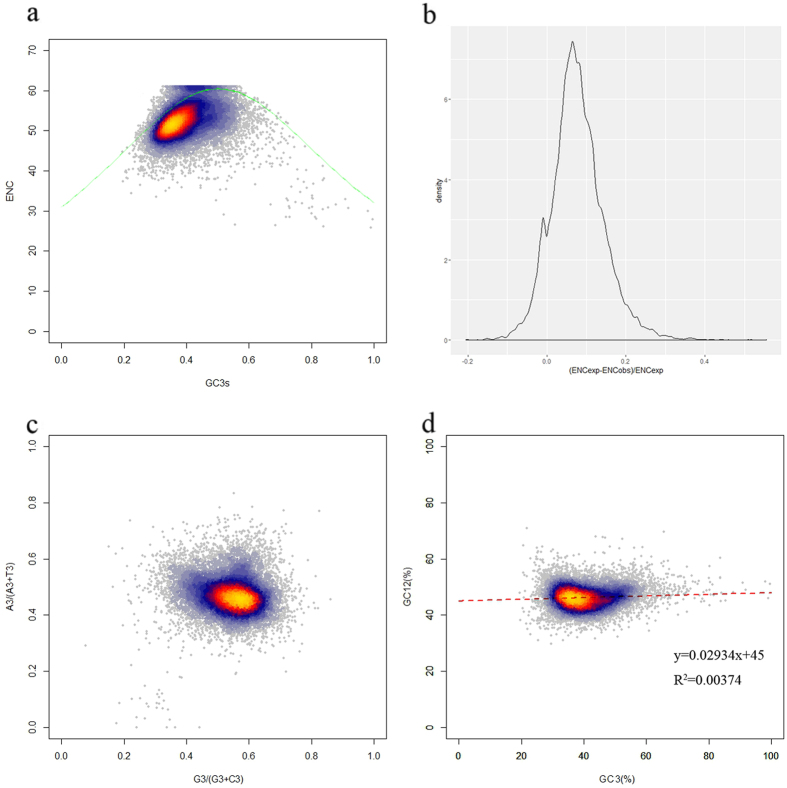
(**a**) ENC–GC3 plot. ENC represents the effective number of codons, and GC3 is the GC content of synonymous codons at the third position. The solid line represents the expected curve when codon usage bias is only affected by mutation pressure. (**b**) Frequency distribution of effective number of codons (ENC). (**c**) PR2-bias plot [A3/(A3 + U3) against G3/(G3 + C3)] of *G. biloba* four-fold degenerate codons. (**d**) Neutrality plot analysis of GC12 and GC3 contents. GC12 in this regression plot represents the average value of GC contents at the first and second positions in each codon, and GC3 is the GC content at the third position (r = 0.061, p < 0.01).

To better understand the relations between *G. biloba* gene composition and codon usage bias, an ENC–GC3 scatter diagram was constructed (Fig. 2a). This method is usually used to estimate the important factors in the formation of codon usage pattern. The average GC3 content was 39.99% and the value was lower than the total average GC content (44.81%). When codon usage pattern is only affected by GC3 resulting from mutation pressure, the expected ENC values should be just on the solid curved line shown in Fig. 2a. In the figure, though some dots were on or very close to the curve, most points were far from the curve, indicating strong bias. The results revealed that codon usage bias tended to be stronger when GC contents were higher. Although mutation pressure may be a factor in the formation of *G. biloba* codon usage bias, some independent factors, such as natural selection strongly affected the bias pattern and these factors would be much more important than the obvious mutation pressure.

To obtain a more accurate estimation of the differences in ENC values, the results of (ENCexp – ENCobs)/ENCexp were calculated (Fig. 2b). The frequencies of unigenes were highest when the value was within 0.00–0.05. The frequencies were nearly the same for the two intervals of −0.05 to 0.00 and 0.05 to 0.10. The results showed that most observed ENC values were lower than expected values, although in most cases the differences were not significant and the results provided more evidence for the existence of other factors that affected the formation of *G. biloba* codon usage bias.

### Parity rule 2 bias plot demonstrates G/A are more frequent

Four-fold degenerate codons are available for the estimation of neutral mutation rate. If codon usage bias is only caused by mutation pressure, AU or GC should be used equally among the degenerate codon groups in a gene^28^. Meanwhile, natural selection for codon selection would not necessarily cause proportional use of G and C (A and U)^29^,^30^. The four-codon amino acids are alanine, glycine, proline, threonine, valine, arginine (CGA, CGU, CGG and CGC), leucine (CUA, CUU, CUG and CUC) and serine (UCA, UCU, UCG and UCC). The results showed that G and A were used more frequently than C and U in *G. biloba*, which contradicted the angiosperms results – although some points on the figure showed proportionality, most did not (Fig. 2c). This observation also indicated that natural selection pressure might play a major role in *G. biloba* codon usage bias, and mutation pressure would be a minor factor.

### Natural selection is the dominant influence on *G. biloba* codon usage

Although the ENC–GC3 plot and the above analysis reflected the main factors that influenced codon usage bias, they did not estimate precisely which of mutation pressure or natural selection was more important. Then a neutrality plot was constructed based on the GC3 and GC12 contents of *G. biloba* (Fig. 2d). The GC3 range of *G. biloba* was in a very wide range (0.195–0.996), while GC1 and GC2 contents were relatively narrow (0.254–0.756 and 0.207–0.865). The correlation between GC1 and GC2 was very strong (r = 0.998, p < 0.01), while neither GC1 nor GC2 showed significant correlation with GC3, suggesting that codons were affected by mutation pressure to a very limited degree. In addition, the slope of the neutrality plot revealed that mutation pressure only accounted for 2.93% of *G. biloba* codon usage, and natural selection together with some other minor factors accounted for the remaining 97.07%^24^. The results demonstrated that natural selection played a very important or even a dominant role in the formation of *G. biloba* codon usage.

### GC contents obviously differ in high/low-expressed unigenes

**Figure 3:**
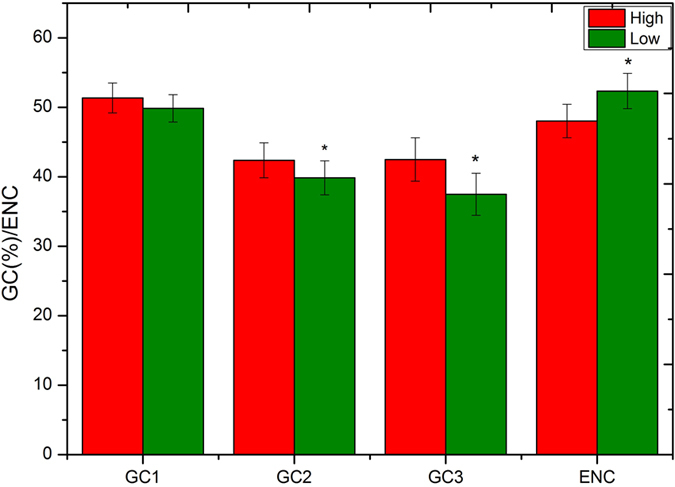
GC content and codon usage bias variation among high and low-expression unigenes. Each 1,500 unigenes with highest and lowest FPKM values were regarded as high/low datasets respectively. Asterisk represents p < 0.05 by t-test.

In next-generation sequencing technology, Fragments Per Kilo base of exon per million fragments Mapped (FPKM) is currently the most widely used parameter for the estimation of gene expression; and previous qRT-PCR results on unigenes of *G. biloba* were consistent with the FPKM values^31^ (Supplementary File 2). As a result, we replaced the codon adaptation index (CAI), which is widely used in the analysis of codon usage bias, with FPKM for the estimation of gene expression. The 1,500 unigenes with highest and lowest FPKM values were set as high/low-expression unigenes, respectively. To avoid the problem of low-expression values being affected by sequencing instrument errors, PCR bias and some other contaminants, unigenes with FPKM <1.0 were excluded from the dataset. Compared to low-expression dataset, mean GC1, GC2 and GC3 all rapidly increased in high-expression dataset together with more codon usage bias (Fig. 3). GC3 had the most significant variation in both two datasets (SD = 8.46 and 9.53), and the variation in GC2 and GC1 was relatively lower but still significant to GC2. Although there were no correlations between GC1/GC2 and GC3, the rapid increase in these datasets suggested that gene expression was not only affected by codon usage bias, but also by amino acid composition.

### Correspondence analysis

**Figure 4:**
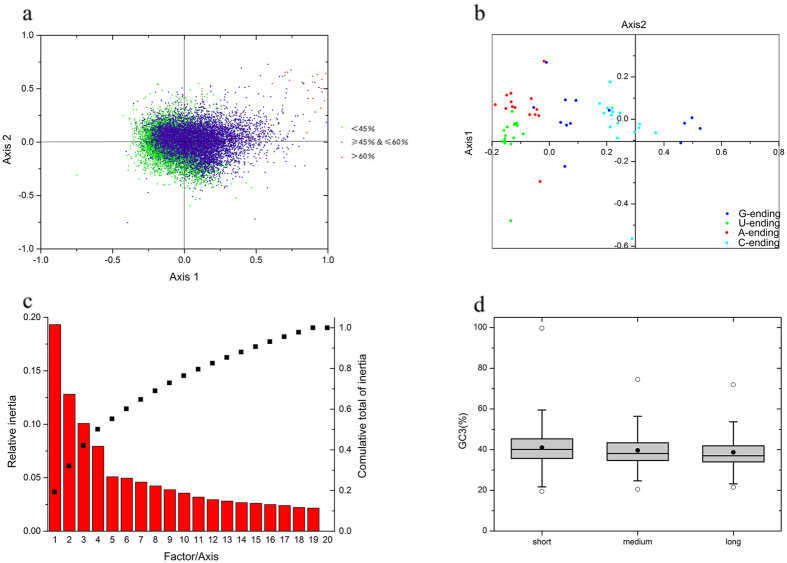
(**a**) Correspondence analysis of various GC content unigenes based on RSCU values. (**b**) Correspondence analysis of codons ending with different codons based on RSCU values. (**c**) The relative and cumulative inertia of the first 20 factors from correspondence analysis based on the amino acid usage frequencies. (**d**) Box plot of GC content variation with different protein length. The dark dots represents the mean, the bottom and top of the box were the lower and upper quartiles, respectively, and the ends of the whiskers were the lowest and highest data still within 1.5 times the interquartile range of the lower and higher quartiles, respectively.

To further investigate the variation of synonymous codon usage pattern in *G. biloba*, correspondence analysis was conducted using the RSCU values and amino acid usage respectively. When the COA was applied to RSCU values, the first two axes accounted for 15.05% and 10.38% of the total variation, respectively. A scatter plot of these axes is shown in Fig. 4a, and the distance in the figure represents the variation of different unigenes’ RSCU values. Unigenes with different GC contents were labeled with different colors, so as to investigate the effects of GC contents on codon usage bias. The unigenes with different GC contents were distributed along the primary axis, and the correlation analysis results revealed that unigene position on the primary axis was significantly positively correlated with ENC (r = 0.365, p < 0.01) and axis 2 (r = 0.879, p < 0.01) values. Codons with GC < 45% and GC of 45–60% didn’t separate very clearly (Fig. 4a).

Then all 59 synonymous codons were divided into four categories: A- U-, G– and C-ending codons (Fig. 4b). A/U-ending codons were more concentrated compared to G/C-ending codons together with a more similar bias pattern. Consistent to parity rule 2 plot results, G-ending codons were more prevalent than C-ending codons, and A-ending codons were more frequent than U-ending codons. Previous studies in angiosperms revealed higher usage frequencies for U/C-ending compared to A/G-ending codons^32^, and our result for gymnosperms differed.

When the COA was applied to amino acid usage, the number of axes generated was 20 and the first four accounted for 50.15% of the total variation. The primary axis accounted for 19.32% and the second axis for 12.81% (Fig. 4c). The primary axis showed a very weak correlation with ENC values (r = 0.074, p < 0.01), and the correlation between the primary axis and GC3 was significant (r = 0.268, p < 0.01). In addition, correlations of the primary axis with the general average hydropathicity (GRAVY) score and with the aromaticity (Aromo) score were significant (r = 0.465, p < 0.01; r = 0.396, p < 0.01; respectively). Studies on *E. coli* have shown that the most important trend of amino acid usage is hydrophobicity, and the second trend is aromatic acid^33^. The above analysis indicated that there was a high probability that translation efficiency in *G. biloba* was influenced by the selection of amino acids and codon usage bias.

### Analysis results of protein length and other factors associated with codon usage bias

Correlation analysis was performed for ENC and the first axis position with protein length – both factors were negatively correlated with protein length (r = −0.20 and −0.184, p < 0.01, respectively). This suggested that unigenes with strong codon usage bias tended to have longer protein length. However, most previous studies demonstrated that sequences with shorter length tended to have stronger codon usage bias, which contradicted our study results and the results in *Picea* family^34^. To confirm our results, all available full-length cDNA sequences of *G. biloba* from online databases were collected and analyzed independently. The correlation analysis between lengths of these sequences and ENC values further validated our result (r = −0.261, p < 0.01). The sequences were then subdivided into three categories (300–900, 900–1800 and >1800 bp) and GC contents at three sites were calculated respectively (Fig. 4d). The results demonstrated that coding sequences with different lengths had very similar GC1 and GC2 values, while GC3 values showed a declining trend towards shorter sequences.

Additionally, there were significant positive correlations between GRAVY and ENC values, and between Aromo and ENC values, although both were very weak (r = 0.194 and 0.190, respectively, p < 0.01). This suggested that codon usage bias in *G. biloba* was to some extent related to hydrophobic values and aromaticity.

### Determination of optimal codons

Each 5% of high- and low-expressed unigenes were selected as datasets respectively. The average RSCU values of the high/low-expressed datasets are shown in Supplementary File 3. When one codon’s RSCU values in both two datasets were significantly correlated by a two-way Chi-square test (p < 0.01), the codon was then defined as optimal. In *G. biloba*, there were 18 optimal codons verified, and 11 of them ended with A/U (Supplementary File 3). Compared to RSCU results, the proportion of G/C-ending codons was significantly greater.

### Codon usage patterns comparisons among unigenes with different functions

**Figure 5:**
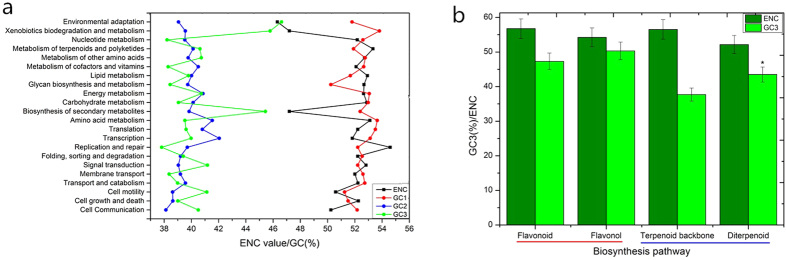
(**a**) GC content and codon usage bias variation among unigenes with different functions based on KEGG annotation. (**b**) Variation of GC content and codon usage bias in the biosynthesis pathways of flavonoids and terpenoid backbone. Asterisk represents p < 0.05.

According to the annotation results in the Kyoto Encyclopedia of Genes and Genomes (KEGG) database, 5,164 annotated unigenes (7.53%) were divided into five main categories and 22 subclasses: Cellular Processes (cell communication; cell growth and death; cell motility; transport and catabolism); Environmental Information Processes (membrane transport; signal transduction); Genetic Information Processes (folding, sorting and degradation; replication and repair; transcription; translation); Metabolism (from amino acid metabolism to xenobiotics biodegradation and metabolism) and Organism Systems (environmental adaptation). After the screening procedure, 1,235 unigenes were selected for the subsequent analysis; and the metrics about these transcripts are available in Supplementary File 4. Then mean ENC values and GC contents of these pathways were recalculated respectively (Fig. 5a). The mean GC1 and GC2 of these pathways were similar (SD = 0.84 and 0.90, respectively) while their GC3 and ENC values differed greatly (SD = 2.56 and 1.68, respectively). Pathways involved in environmental adaptation had the highest GC3 (46.8%) together with the strongest codon bias (ENC = 46.5); and the two following pathways were xenobiotics biodegradation and metabolism, and biosynthesis of secondary metabolites respectively. Pathway involved in replication and repair had the lowest GC3 (37.7%) and codon bias (ENC = 54.8). The results showed that the codon usage pattern in *G. biloba* varied across the different functional categories. The pathways involved in processes of environmental interaction tend to have higher GC3 contents and codon usage bias.

To better understand the reason for the high GC3 and strong codon usage bias in biosynthesis of secondary metabolites pathway, four detailed KEGG synthetic pathways of two richest secondary metabolites (flavonoids and terpene lactones) in *G. biloba* were then selected and analyzed: terpenoid backbone biosynthesis and diterpenoid biosynthesis (downstream); and flavonoid biosynthesis and the downstream flavones and flavonol biosynthesis. Although mean GC1 and GC2 for the two groups didn’t vary greatly, mean GC3 and codon usage bias for both were higher in the downstream comparing to the upstream pathway (Fig. 5b).

### Codon usage patterns comparison in different gymnosperms

**Figure 6:**
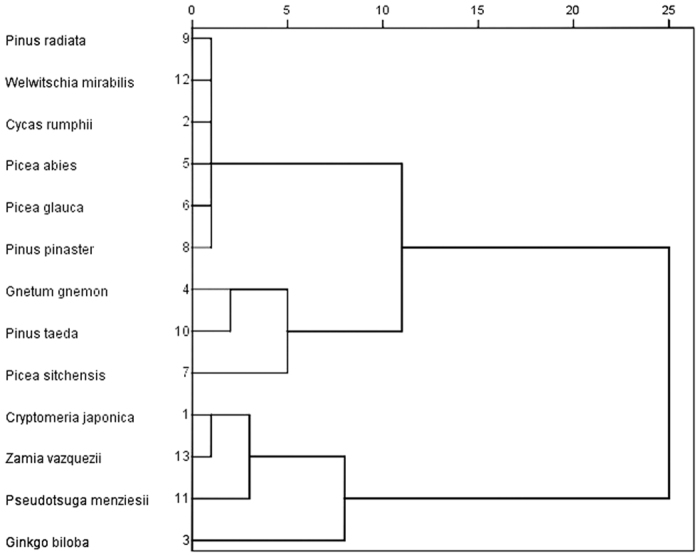
Dendrogram of 13 gymnosperms according to GC3 values.

In addition to *G. biloba*, 12 gymnosperms in four orders were collected and analyzed: Coniferales, Cycadales, Ginkgoales and Gnetales. Their ENC values varied from 47.89 (*Cycas rumphii*) to 53.64 (*Zamia fischeri*) with SD of 1.62, with the ENC of *G. biloba* the second highest (52.66). The overall codon usage bias among gymnosperms was similar, although GC3 varied widely. GC3 of these species ranged from 39.02% (*G. biloba*) to 45.89% (*Pinus radiata*). The clustering dendrogram of these species based on GC3 was partial consistent with the custom classification (Fig. 6).

## Discussion

### Reliability of data and selection of expression criteria

Although next-generation sequencing technology has greatly enhanced the quantity and quality of sequencing data, and the 25.08G of clean data in our study meets the required *G. biloba* transcriptome sequencing depth, the lack of a reference genome is still a limiting factor for our analysis. To minimize the potential error, only coding sequences ≥300 bp annotated as unigene with ≥more than 90% identity compared to the Nr database using blastx were selected-hence one unigene finally corresponded to only one CDS and all unsuitable unigenes or CDSs were discarded. For some similar sequences that might have resulted from alternative splicing or multi-copy genes, we usually referred to annotation results in databases and excluded all suspicious sequences.

In our research, FPKM applied from high-throughput sequencing technology was set as the criterion^35^. In most previous analysis of codon usage pattern, CAI was used for estimation of gene expression. CAI ranges from 0 to 1.0, with higher value representing stronger expression^36^. In contrast, FPKM means the counts of fragments annotated as one unigene per unit length, and then the relative expression is estimated. Unlike the very extreme codon usage bias pattern in prokaryotes, selection of synonymous codons in eukaryotes is much more complex. The datasets for calculating CAI are originally from <30 highly expressed genes, although the algorithm has been modified several times, the datasets are still derived from prokaryotes and lower eukaryotes. Consequently, we concluded that it was not appropriate to apply CAI directly to estimating gene expression of higher eukaryotes. Three different types of CAI calculation software were used^37^,^38^,^39^ and correlations calculated between their resulting CAI values and normalized *G. biloba* FPKM values-no significant results were found for any CAI software (Supplementary File 5). However, it should be noted that FPKM is a poor measure when performing differential expression analysis^40^ and may not be adequate to compare expression across samples. Hence RSEM was used to quantify transcript expression before they were transformed to FPKM^41^. In addition, unigenes with extreme low FPKM values (<1.0) were excluded to avoid the bias caused by low-expressed unigenes. Three technical replicates were calculated for each sample and the bowtie parameter was set as mismatch 2. Since our previous experiments validated that FPKM values were similar to qRT-PCR results, we concluded that FPKM was more suited than CAI to directly estimate gene expression in higher eukaryotes, despite CAI being widely used in many previous studies.

### Characteristics of *G. biloba* codon usage pattern

The overall codon bias pattern in *G. biloba* tended to use A/U -ending codons, and among all 28 preferred codons in *G. biloba* (RSCU > 1.0), 23 ended with A/U. The proportion was much higher than that in other known species: there are usually about 23 preferred codons in dicots and about 16 of them end with A/U, and about 22 preferred codons in monocots with less than five A/U-ending codons^42^,^43^. Progressing through species from gymnosperms to dicots, then to monocots, there was an increase in G/C-ending codons, and this might reflect the trend of evolution on codon usage bias from A/U to G/C. Additionally, among 18 amino acids encoded by synonymous codons, A-ending codons were more common than U-ending codons, and G-ending codons were more common than C-ending. This result was not consistent with the analysis in dicots and monocots^44^. In addition, 18 codons in *G. biloba* were defined as optimal and 11 of them were ended with A/U. In microorganisms, studies have shown that optimal codons can effectively regulate the folding and elongation rate of proteins, thus significantly promoting synthesis of proteins^45^,^46^. These optimal codons could be beneficial in subsequent studies on degenerate primers and protein synthesis rate in higher eukaryotes.

Associated with codon usage bias, GC3 in higher plants also showed some patterns. There was an obvious enrichment of GC3 contents proceeding from gymnosperms to monocots. For example, GC3 content in *Zea mays* was 58.6%, 42.01% in *Glycine max*, 40.61% in *Arabidopsis thaliana*, and 39.02% in *G. biloba*^47^. Comparison between GC3 contents in different gymnosperms revealed that although GC3 were similar between close species, the clustering result based on GC3 was not highly consistent with the custom classification, suggesting that GC3 might be affected by independent factors in different species.

### Is there a trend from A/U- to G/C-ending codons in *G. biloba*

Although there were more codons ending with A/U than G/C according to RSCU values, the comparison results revealed that GC3 was much higher in high- expression unigenes (45.70% vs. 37.98%). In addition, the proportion of optimal codons ending with G/C was larger than that in RSCU values, and the correlation between FPKM values and GC3 was significant and positive (r = 0.286, p < 0.01). There seemed to be a trend for *G. biloba* to have a codon bias for A/U- over G/C-ending, consistent with previous results in *Picea* gene families^34^. It is noteworthy that although GC1/GC2 was not correlated with GC3, they had higher values in high- than low-expression datasets, suggesting that both codon usage bias and amino acid composition was associated with gene expression.

The comparison between genomic and genic GC contents indicated a remarkable difference in genic GC contents (44.81% vs. 35.63%). Even though mean GC3 was lower than mean GC1 and GC2, it was still higher than average GC contents. This result also supports our assumption: lower genomic GC contents contributed to the trend of A/U-ending codons in preferred codons, while optimal codons and high-expression unigenes in *G. biloba* were more likely to have G/C-ending codons. One problem was that with the lack of a whole *G. biloba* genome, estimation of genomic GC contents was based on the random genomic high-throughput sequencing data which was previously sequenced in our laboratory using Roche 454 sequencing system, and we think >300,000 sequences of ≥500 bp sequences generated from that platform would be needed to accurately estimate the mean genomic GC content in *G. biloba*.

### Codon usage pattern for different function categories

According to the annotation results based on the KEGG database, mean GC1 and GC2 didn’t vary greatly across the different function categories, while GC3 contents significantly differed. Mean GC3 contents were highest in environmental adaptation, xenobiotics biodegradation and metabolism, and biosynthesis of secondary metabolites together with the most biased codon usage. We presume that richer and more stable GC contents enabled better adaptation to the environment. To determine why unigenes involved in biosynthesis of secondary metabolites had such high GC3 contents, pathways involved in the two most important and richest secondary metabolic compounds in *G. biloba* were selected and analyzed: flavonoids and diterpene backbone. The results indicated that GC3 tended to increase from upstream to downstream synthetic processes together with a stronger codon bias. Although the differences along the steps were not significant, the environment was likely the major cause for GC3 variation among different function categories and adapting genes seemed to be the major reason for GC3 enrichment in *G. biloba*. This may explain why pathways involved in environment adaptation contained higher GC3 contents. Since the synthetic processes of secondary metabolites are usually more time-consuming and complex, there is more possibility that they will be affected by the external environment, hence the higher GC3 content downstream in the synthetic pathway.

### Formation of *G. biloba* codon usage pattern

The formation of codon usage bias is affected by many factors, and two generally accepted major forces are mutation pressure and natural selection. The neutrality plot is the most widely used method to accurately estimate the balance between mutation pressure and natural selection. When the codon usage bias is only affected by mutation pressure, the quantitative relation between GC3 and GC12 should be nearly equal, and the slope would be 1. Most previous studies in plants revealed that natural selection was more important than mutation pressure, and GC1/GC2 usually positively correlated with GC3^24^,^48^. In our study, GC1/GC2 had no correlation with GC3 and the slope of neutrality plot was only 0.026-much lower than found in *Oryza sativa* (0.143) and *Zea mays* (0.140)^43^, -these results showed that natural selection was dominant in *G. biloba*. Therefore, the long period of evolution lasting for more than 280 million years, the codons of *G. biloba* were majorly affected by natural selection, and it seems that GC3 had nearly successfully escaped from mutation pressure restriction. In *G. biloba*, the extremely long time and intensive natural selection resulted in the present codon usage pattern.

## Materials and Methods

### Details of plant material and transcriptome sequencing

*G. biloba* kernels were used for transcriptome sequencing. Seeds from a mature *Ginkgo* tree growing at Nanjing Forestry University were collected at five developmental time points (8 July: Gb_Seed 1; 5 August: Gb_Seed 2; 2 September: Gb_Seed 3; 20 November: Gb_Seed 4; 2 December: Gb_Seed 5). After the removal of the testae, three samples of kernels at each time point were prepared for sequencing.

Before RNA extraction, RNA degradation and contamination were monitored on 1% agarose gels. RNA integrity was assessed using the RNA Nano 6000 Assay Kit in an Agilent Bioanalyzer 2100 system (Agilent Technologies, Palo Alto, CA, USA). A total of 3 μg RNA was used as input per sample. Clustering of the index-coded samples was performed on a cBot Cluster Generation System using the TruSeq PE Cluster Kit v3-cBot-HS (Illumina) according to the manufacturers’ instructions. After cluster generation, the library preparations were sequenced on an Illumina Hiseq 2000 platform, and paired-end reads were generated.

Raw transcripts in FASTQ format generated by platform were processed using in-house Perl scripts. Then clean data were obtained by removing adapter-containing, poly-N and low-quality reads from the raw data. Trinity (version: v2012-10-05) was used for the assembly^31^,^49^. Parameters were set as default, except that min_kmer_cov was set as 2. A total of 25.08 Gb of sequencing data were generated, comprising 185,075,486 raw reads and 167,215,904 clean reads. The base average error rate was 0.08%, and the average Q20 and Q30 values were 95.24% and 87.12%, respectively. A total of 112,946 transcripts were generated with average length 1,262 bp, and N50 of 2,269 bp. The raw data were submitted to NCBI database (accession number: SRP 062414).

### Sequence information and annotation

Coding sequences (CDSs) were predicted based on NR and Swissprot databases. The obtained unigenes were searched against the above databases accordingly initially using blastx (E-value = 1e^−5^). If the unigenes were aligned successfully, open reading frames of transcripts were extracted from the results, and then the CDSs were translated into amino acid sequences according to the standard codon usage table (from 5′ to 3′). To ensure the quality of sequences, low-quality sequences with lengths shorter than 300 bp, with any unidentified bases or internal stop codon included sequences were deleted. The initial alignment results were further verified using EMBOSS: getorf (http://emboss.bioinformatics.nl/cgi-bin/emboss/getorf), and were manually screened to remove those sequences in which the initiation codon could not be recognized or that contained internal gaps. The assembled unigenes were then searched against the KEGG database to find and predict functional classifications and molecular pathways. The EST sequences of 12 gymnosperms were downloaded from PlantGDB (http://www.plantgdb.org) and GenBank respectively (https://www.ncbi.nlm.nih.gov/genbank).

### Codon usage indices

After the screening of sequences, some basic indices of codon usage bias were calculated, such as the base composition at the first/second/third site of codons (GC1/GC2/GC3), the RSCU for each gene.

If all codons were used with equal frequencies, then the RSCU statistics could be calculated by dividing the observed usage of a codon^50^, using the formula below:


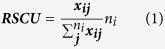


where *x*_*ij*_ represents the frequency of codon *j* encoding for the *i*^th^ amino acid and *n*_*i*_ represents the number of synonymous codons encoding the *i*^th^ amino acid. Codons with RSCU >1.0 occur when they are used with higher frequencies than random, and RSCU <1.0 means the opposite^51^.

The ENC was calculated to estimate synonymous codon bias extent of a single gene, and is a relatively direct method to also evaluate codon usage bias^52^. The formula of ENC is:


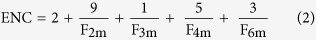


In this formula, F_km_ (k = 2, 3, 4, and 6) is the mean of F_k_ values for the k-fold degenerate amino acids and F_k_ is estimated using the following formula:


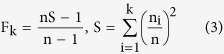


where n_i_ is the total number of the *i*^th^ codon for that amino acid and n is the total number of occurrence of the codons for that amino acid.

When the codon usage is only affected by mutation pressure, the formula of expected ENC value is given by:


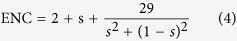


s represents the value of (G + C) 3%.

Ideally the ENC should be in the range of 20–61. This means that when each amino acid is encoded by only one codon, ENC should be 20, and when all synonymous codons have an equal chance, ENC should be 61. The more significant the codon usage bias, the lower the ENC values.

The general average hydropathicity score (GRAVY) is for the hypothetical translated gene product. GRAVY is calculated as the arithmetic mean of the sum of the hydropathic indices of each amino acid^53^. This index has been used to quantify the major correspondence analysis (COA) trends in the amino acid usage of *Escherichia coli* genes^33^. The aromaticity score (Aromo) defines the frequency of aromatic amino acids (Phe, Tyr and Trp) in the hypothetical translated gene product. According to Lobry’s research, the strongest trend in the variation in the amino acid composition of *E. coli* genes is correlated with GRAVY, the second trend is correlated with gene expression, while the third is correlated with Aromo^33^. The variation in amino acid composition can have applications for the analysis of codon usage.

### Neutrality plot

The neutrality plot was used to estimate the most important factors influencing codon usage bias between mutation pressure and natural selection^24^. The closer the plot slope is to 0, the less influence there is on codon usage from directional mutation pressure. When the slope is 1, this indicates that codon usage bias is totally formed by directional mutation pressure and shows complete neutrality. GC1 and GC2 contents were calculated with Perl scripts.

### Determination of optimal codons

Based on the calculated results, 5% of genes with extreme high and low FPKM values were regarded as two datasets (high and low expression, respectively). A two-way Chi-square test was used, and when the RSCU value of one codon in the high-expression dataset was significantly correlated with that in the low-expression dataset (p < 0.01), the codon was defined as optimal^54^.

### Correspondence analysis

COA is widely used to study the correlation between codon usage and other factors^42^. It is conceptually similar to principal component analysis but it applies to categories instead of continuous data. COA creates a series of orthogonal axes to identify trends that explain the data variation, with each subsequent axis explaining a decreasing amount of the variation. COA positions each gene and codon (or amino acid) on these axes. An important property is that the ordination of the rows (genes) and columns (codons or amino acids) are super-imposable. In this analysis, 59 codons (excluding Met, Trp and three stop codons) were placed in a multi-dimensional space, and they were used to determine all the major factors affecting codon usage bias of *G. biloba*.

### Statistical analysis

Correlation analysis and t-tests were conducted using SPSS 19.0 (SPSS Inc. software, Chicago, Il, USA) and the statistical approach was Spearman’s rank; Origin 9.0 was used for the figures. CodonW (Ver.1.4.2) (http://mobyle.pasteur.fr/cgi-bin/portal.py#forms::CodonW), CHIPS (http://mobyle.pasteur.fr/cgibin/portal.py#forms::chips) and CUSP (http://mobyle.pasteur.fr/cgi-bin/portal.py#forms::cusp) were mainly used to calculate the indices of codon usage bias.

## Additional Information

**How to cite this article**: He, B. *et al*. Analysis of codon usage patterns in *Ginkgo biloba* reveals codon usage tendency from A/U-ending to G/C-ending. *Sci. Rep.* **6**, 35927; doi: 10.1038/srep35927 (2016).

**Publisher’s note**: Springer Nature remains neutral with regard to jurisdictional claims in published maps and institutional affiliations.

## Supplementary Material

Supplementary Information

## Figures and Tables

**Table 1:** Codon usage pattern of *G. biloba* genes; the preferred codons are in bold (RSCU > 1.0).

AA	Codon	RSCU	AA	Codon	RSCU
Ala	**GCU**	1.36	Leu	**UUG**	1.55
	GCC	0.71		**CUU**	1.36
	**GCA**	1.61		CUC	0.71
	GCG	0.32		CUA	0.57
Arg	CGU	0.67		**CUG**	1.01
	CGC	0.46	Lys	AAA	0.99
	CGA	0.71		**AAG**	1.01
	CGG	0.55	Phe	**UUU**	1.20
	**AGA**	2.04		UUC	0.80
	**AGG**	1.57	Pro	**CCU**	1.46
Asn	**AAU**	1.31		CCC	0.68
	AAC	0.69		**CCA**	1.48
Asp	**GAU**	1.38		CCG	0.39
	GAC	0.62	Ser	**UCU**	1.48
Cys	**UGU**	1.03		UCC	0.79
	UGC	0.97		**UCA**	1.37
Gln	**CAA**	1.03		UCG	0.41
	CAG	0.97		**AGU**	1.07
Glu	**GAA**	1.10		AGC	0.88
	GAG	0.90	Thr	**ACU**	1.30
Gly	**GGU**	1.06		ACC	0.72
	GGC	0.79	Tyr	**ACA**	1.57
	**GGA**	1.37		ACG	0.41
	GGG	0.78		**UAU**	1.26
His	**CAU**	1.33		UAC	0.74
	CAC	0.67	Val	**GUU**	1.45
Ile	**AUU**	1.45		GUC	0.64
	AUC	0.74		GUA	0.74
	AUA	0.81		**GUG**	1.17
Leu	UUA	0.80			

AA: amino acids.
