# Supplementary material for: Analysis of codon usage patterns in Ginkgo biloba reveals codon usage tendency from A/U-ending to G/C-ending
Source: Sci Rep. 2016 Nov 3;6:35927. doi: 10.1038/srep35927 (PMC5093902; doi:10.1038/srep35927)
Supplement: Supplementary Information [file srep35927-s1.doc]

**Analysis of codon usage patterns in *Ginkgo biloba* reveals codon usage tendency from A/U-ending to G/C-ending**

**Bing He, Hui Dong, Cong Jiang, Fuliang Cao, Shentong Tao, Li-an Xu**

**Supplementary File 1: GC content of *Pinus taeda* and *Picea abies***

| **Species** | **Genic GC content (%)** | **UTR GC content (%)** |
| --- | --- | --- |
| *Pinus taeda* | 50.62 | 47.28 |
| *Picea abies* | 45.46 | 42.88 |

**Supplementary File 2: qRT-PCR results on unigenes of *G. biloba***

**
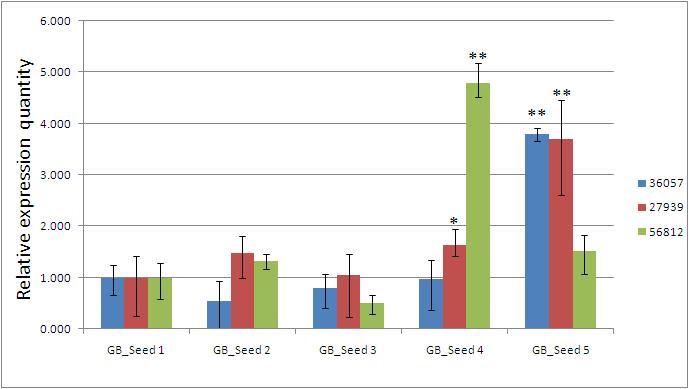
**

The unigene IDs and their log_2_(fold change) values at different time points are listed below:

com36057_c0 (0-0-1.79-2.31);

comp27939_c0(0-0-1.32-3.10);

comp56812_c0 (0-0-3.54-2.18);

**Supplementary File 3: Translational optimal codons of *G. biloba***

AA: amino acids; N: number of codons. Optimal codons were determined through Chi-square test. Asterisk represents p < 0.01.

| AA | Codon | High | Low | AA | Codon | High | Low |
| --- | --- | --- | --- | --- | --- | --- | --- |
|  |  | RSCU(N) | RSCU(N) |  |  | RSCU(N) | RSCU(N) |
| Phe | UUU | 1.46(7590) | 0.78(3920) | Ser | UCU* | 1.79(8466) | 1.05(3572) |
|  | UUC | 0.54(2799) | 1.22(6095) |  | UCC | 0.52(2464) | 1.31(4458) |
|  | UUA | 1.02(4576) | 0.44(1671) |  | UCA* | 1.69(7996) | 0.91(2397) |
|  | UUG* | 1.62(7253) | 1.07(4774) |  | UCG | 0.20(967) | 1.02(3460) |
|  | CUU* | 1.62(7264) | 0.91(3413) |  | AGU | 1.20(5653) | 0.87(2262) |
|  | CUC | 0.38(1705) | 1.45(5475) |  | AGC | 0.59(2769) | 1.24(4193) |
|  | CUA | 0.66(2967) | 0.36(1363) | Pro | CCU* | 1.75 (6588) | 1.01 (3173) |
|  | CUG | 0.69(3094) | 1.56(5884) |  | CCC | 0.39 (1463) | 1.16 (3637) |
| Ile | AUU | 1.62(7662) | 1.16(4481) |  | CCA* | 1.68 (6326) | 0.84 (2636) |
|  | AUC | 0.50(2347) | 1.17(4516) |  | CCG | 0.17 (653) | 0.99 (3126) |
|  | AUA | 0.88(4170) | 0.67(2566) | Thr | ACU* | 1.55 (5661) | 0.84 (2557) |
| Val | GUU | 1.69(8259) | 1.24(4011) |  | ACC | 0.43 (1565) | 1.29 (3928) |
|  | GUC | 0.46(2226) | 1.04(4022) |  | ACA* | 1.83 (6688) | 0.90 (2726) |
|  | GUA | 0.85(4179) | 0.54(1687) |  | ACG | 0.20 (714) | 0.97 (2954) |
|  | GUG* | 1.80(4898) | 1.19(5747) | Ala | GCU* | 1.60 (8853) | 0.86 (3808) |
| Tyr | UAU* | 1.48(5342) | 0.87(2676) |  | GCC | 0.43 (2402) | 1.36 (6012) |
|  | UAC | 0.52(1883) | 1.13(3474) |  | GCA* | 1.83 (10160) | 0.94 (4161) |
| His | CAU | 1.54(6021) | 1.26(2887) |  | GCG | 0.14 (784) | 0.83 (3647) |
|  | CAC | 0.46(1815) | 1.04(3144) | Cys | UGU* | 1.35 (3639) | 0.66 (1836) |
| Gln | CAA | 1.09(7672) | 0.80(3491) |  | UGC | 0.65 (1760) | 1.34 (3689) |
|  | CAG | 0.91(6405) | 1.20 (5280) | Arg | CGU | 0.86 (2413) | 0.59 (1478) |
| Asn | AAU | 1.50 (9548) | 1.07 (4722) |  | CGC | 0.34 (955) | 0.89 (2216) |
|  | AAC | 0.50 (3170) | 1.03 (5060) |  | CGA | 0.78 (2192) | 0.70 (1737) |
| Lys | AAA | 1.03 (9126) | 0.81 (4949) |  | CGG | 0.54 (1537) | 0.75 (1869) |
|  | AAG* | 1.97 (8644) | 1.19 (7316) |  | AGA | 1.96 (5539) | 1.63 (4069) |
| Asp | GAU | 1.51 (12402) | 1.17 (5065) |  | AGG* | 1.52 (4290) | 0.93 (3568) |
|  | GAC* | 1.13 (4020) | 0.48 (5350) | Gly | GGU | 1.33 (7085) | 0.76 (3243) |
| Glu | GAA | 1.16 (12739) | 0.88 (5428) |  | GGC | 0.57 (3046) | 1.28 (5465) |
|  | GAG* | 1.84 (9297) | 1.12 (6968) |  | GGA | 1.41 (7483) | 1.11 (4731) |
|  |  |  |  |  | GGG* | 1.69 (3659) | 0.85 (3602) |

**Supplementary File 4:** Metrics of the unigenes annotated with the KEGG database

| **KEGG annotation** | **GC content (%)** | **Mean length (bp)** |
| --- | --- | --- |
| Environmental adaptation | 45.15 | 1,125 |
| Xenobiotics biodegradation and metabolism | 46.80 | 897 |
| Nucleotide metabolism | 43.79 | 465 |
| Metabolism of terpenoids and polyketides | 45.07 | 1,062 |
| Metabolism of other amino acids | 45.12 | 1,350 |
| Metabolism of cofactors and vitamins | 44.57 | 1,025 |
| Lipid metabolism | 44.87 | 927 |
| Glycan biosynthesis and metabolism | 43.65 | 792 |
| Energy metabolism | 45.81 | 924 |
| Carbohydrate metabolism | 45.05 | 768 |
| Biosynthesis of secondary metabolism | 46.52 | 987 |
| Amino acid metabolism | 45.51 | 1,122 |
| Translation | 45.19 | 1,110 |
| Transcription | 45.80 | 1,284 |
| Replication and repair | 43.83 | 1,030 |
| Folding, sorting and degradation | 44.54 | 895 |
| Signal transduction | 44.90 | 976 |
| Membrane transport | 44.65 | 1,023 |
| Transport and catabolism | 44.43 | 789 |
| Cell motility | 44.08 | 1,071 |
| Cell growth and death | 43.99 | 1,154 |
| Cell communication | 44.16 | 1,021 |
| Others | 45.23 | 987 |

**Supplementary File 5-1: Correlation analysis between three available CAI software and FPKM values (in-built parameters were applied)**

| unigene ID | gene length | lg(FPKM) | wCAI | cCAI | dCAI |
| --- | --- | --- | --- | --- | --- |
| comp40512_c0 | 1230 | 1.063 | 0.653 | 0.102 | 0.841 |
| comp28205_c0 | 566 | 0.319 | 0.730 | 0.091 | 0.814 |
| comp48762_c0 | 3651 | 1.629 | 0.689 | 0.109 | 0.849 |
| comp46484_c0 | 1193 | 2.289 | 0.707 | 0.093 | 0.816 |
| comp47167_c0 | 2596 | 2.271 | 0.687 | 0.091 | 0.824 |
| comp38288_c0 | 917 | 1.570 | 0.817 | 0.121 | 0.813 |
| comp50998_c0 | 4639 | 0.996 | 0.671 | 0.106 | 0.845 |
| comp42118_c0 | 1831 | 0.276 | 0.689 | 0.112 | 0.833 |
| comp48668_c1 | 1658 | 1.435 | 0.647 | 0.102 | 0.820 |
| comp49248_c0 | 2652 | 0.609 | 0.654 | 0.068 | 0.798 |
| comp50394_c0 | 4888 | 1.037 | 0.615 | 0.084 | 0.824 |
| comp49479_c0 | 4777 | 2.086 | 0.689 | 0.129 | 0.852 |
| comp43590_c0 | 1399 | 1.725 | 0.699 | 0.078 | 0.788 |
| comp41697_c0 | 1067 | 1.788 | 0.668 | 0.107 | 0.837 |
| comp51002_c0 | 8307 | 1.160 | 0.71 | 0.148 | 0.823 |
| comp48871_c0 | 2919 | 1.365 | 0.689 | 0.106 | 0.827 |
| comp40229_c0 | 1406 | 1.878 | 0.695 | 0.08 | 0.793 |
| comp47521_c0 | 1983 | 1.110 | 0.714 | 0.096 | 0.805 |
| comp36472_c0 | 976 | -0.080 | 0.709 | 0.109 | 0.825 |
| comp48144_c0 | 1754 | 0.615 | 0.852 | 0.082 | 0.723 |
| comp38221_c0 | 948 | 2.082 | 0.651 | 0.078 | 0.787 |
| comp28542_c0 | 566 | -0.105 | 0.788 | 0.087 | 0.696 |
| comp49659_c0 | 2610 | 0.451 | 0.798 | 0.111 | 0.727 |
| comp50937_c0 | 3049 | 1.187 | 0.703 | 0.121 | 0.847 |
| comp42308_c0 | 1585 | 0.505 | 0.658 | 0.121 | 0.847 |
| comp44845_c0 | 3239 | 0.452 | 0.74 | 0.098 | 0.776 |
| comp46317_c0 | 2019 | 0.489 | 0.723 | 0.088 | 0.804 |
| comp46052_c0 | 1766 | 1.186 | 0.687 | 0.069 | 0.803 |
| comp38220_c0 | 876 | 0.068 | 0.664 | 0.098 | 0.840 |
| comp42795_c0 | 1209 | 0.819 | 0.681 | 0.076 | 0.721 |
| comp44279_c1 | 1973 | 0.435 | 0.713 | 0.042 | 0.702 |
| comp44785_c0 | 1301 | 1.720 | 0.702 | 0.099 | 0.841 |
| comp36843_c0 | 924 | 0.927 | 0.704 | 0.093 | 0.816 |
| comp43302_c0 | 1571 | 0.532 | 0.741 | 0.106 | 0.821 |
| comp36985_c0 | 1277 | -0.007 | 0.659 | 0.118 | 0.845 |
| comp48571_c0 | 1598 | 0.831 | 0.709 | 0.103 | 0.819 |
| comp35344_c0 | 837 | -0.045 | 0.639 | 0.075 | 0.803 |
| comp44013_c1 | 694 | 0.618 | 0.693 | 0.076 | 0.795 |
| comp46751_c0 | 1106 | 1.370 | 0.667 | 0.105 | 0.826 |
| comp39483_c0 | 572 | 0.415 | 0.674 | 0.095 | 0.863 |
| ***correlation***  ***coefficient(r)*** |  |  | ***-0.142*** | ***0.099*** | ***0.249*** |

**5-2 Correlation analysis between three available CAI software** and FPKM values (high FPKM values were set as trainsets)

| unigene ID | lg(FPKM) | wCAI-ref | cCAI-ref | eCAI |
| --- | --- | --- | --- | --- |
| comp40512_c0 | 1.063 | 0.8 | 0.803 | 0.83 |
| comp28205_c0 | 0.319 | 0.828 | 0.828 | 0.846 |
| comp48762_c0 | 1.629 | 0.821 | 0.813 | 0.833 |
| comp46484_c0 | 2.289 | 0.805 | 0.81 | 0.82 |
| comp47167_c0 | 2.271 | 0.805 | 0.804 | 0.817 |
| comp38288_c0 | 1.570 | 0.849 | 0.84 | 0.846 |
| comp50998_c0 | 0.996 | 0.81 | 0.817 | 0.828 |
| comp42118_c0 | 0.276 | 0.807 | 0.808 | 0.828 |
| comp48668_c1 | 1.435 | 0.789 | 0.791 | 0.806 |
| comp49248_c0 | 0.609 | 0.815 | 0.799 | 0.814 |
| comp50394_c0 | 1.037 | 0.795 | 0.797 | 0.807 |
| comp49479_c0 | 2.086 | 0.811 | 0.819 | 0.826 |
| comp43590_c0 | 1.725 | 0.807 | 0.812 | 0.83 |
| comp41697_c0 | 1.788 | 0.792 | 0.798 | 0.817 |
| comp51002_c0 | 1.160 | 0.815 | 0.82 | 0.838 |
| comp48871_c0 | 1.365 | 0.819 | 0.826 | 0.836 |
| comp40229_c0 | 1.878 | 0.815 | 0.816 | 0.84 |
| comp47521_c0 | 1.110 | 0.833 | 0.83 | 0.852 |
| comp36472_c0 | -0.080 | 0.842 | 0.85 | 0.857 |
| comp48144_c0 | 0.615 | 0.843 | 0.838 | 0.865 |
| comp38221_c0 | 2.082 | 0.779 | 0.78 | 0.802 |
| comp28542_c0 | -0.105 | 0.817 | 0.816 | 0.846 |
| comp49659_c0 | 0.451 | 0.87 | 0.861 | 0.882 |
| comp50937_c0 | 1.187 | 0.807 | 0.816 | 0.821 |
| comp42308_c0 | 0.505 | 0.817 | 0.821 | 0.825 |
| comp44845_c0 | 0.452 | 0.822 | 0.823 | 0.833 |
| comp46317_c0 | 0.489 | 0.823 | 0.82 | 0.837 |
| comp46052_c0 | 1.186 | 0.818 | 0.825 | 0.847 |
| comp38220_c0 | 0.068 | 0.83 | 0.834 | 0.855 |
| comp42795_c0 | 0.819 | 0.797 | 0.791 | 0.809 |
| comp44279_c1 | 0.435 | 0.805 | 0.802 | 0.832 |
| comp44785_c0 | 1.720 | 0.816 | 0.817 | 0.823 |
| comp36843_c0 | 0.927 | 0.819 | 0.819 | 0.839 |
| comp43302_c0 | 0.532 | 0.811 | 0.809 | 0.824 |
| comp36985_c0 | -0.007 | 0.815 | 0.81 | 0.824 |
| comp48571_c0 | 0.831 | 0.807 | 0.803 | 0.823 |
| comp35344_c0 | -0.045 | 0.782 | 0.784 | 0.803 |
| comp44013_c1 | 0.618 | 0.826 | 0.825 | 0.829 |
| comp46751_c0 | 1.370 | 0.803 | 0.798 | 0.815 |
| comp39483_c0 | 0.415 | 0.804 | 0.795 | 0.815 |
| ***correlation***  ***coefficient(r)*** |  | ***-0.286*** | ***-0.223*** | ***-0.298*** |
